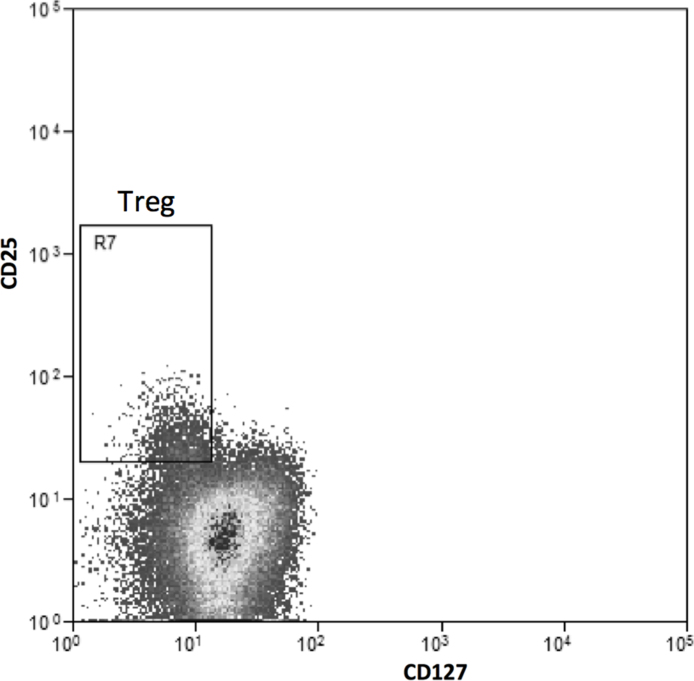# A method for conducting suppression assays using small numbers of tissue-isolated regulatory T cells

**DOI:** 10.1016/j.mex.2014.08.012

**Published:** 2014-08-28

**Authors:** Stephen T. Ward, Ka-Kit Li, Stuart M. Curbishley

**Affiliations:** a National Institute for Health Research (NIHR) Birmingham Liver Biomedical Research Unit (BRU), University of Birmingham, Vincent Drive, Birmingham B15 2TT, UK; b Queen Elizabeth Hospital Birmingham, Mindelsohn Way, Birmingham B15 2WW, UK

**Keywords:** Regulatory T cells, Immunosuppression, Suppression assay, Lymphocyte isolation

## Abstract

The suppression assay is a commonly performed assay, measuring the ability of regulatory T cells (Treg) to suppress T cell proliferation. Most frequently, Treg are obtained from the peripheral blood or spleen. Lower yields are obtained by isolation from other tissues, rendering downstream suppression assays challenging to perform. Furthermore, the importance of suppressive subpopulations of Treg favours their isolation by fluorescent-activated cell sorting. Here we describe a method to isolate Treg from human tissues, using colorectal cancer tissue as an example. Treg suppressive capacity was further examined by expression of CCR5 to demonstrate the ability of our method to assess the suppressive capacity of regulatory T cell subsets.

To optimise the standard suppression assay to achieve our research aims, the following modifications were made:

• Treg, isolated from tissues, were sorted directly into a well-plate.
• Responder T cells, which had been fluorescently-labelled prior to sorting, were added directly into the well-plate.
• Human Treg Suppression Inspector beads (Miltenyi Biotec Ltd., UK) provided a polyclonal stimulus for proliferation and were added to each well at a bead:lymphocyte ratio of 1:2.

This method quantified the suppression of responder T cell proliferation by small numbers of strictly-defined Treg populations isolated from tissues.

## Method details

Regulatory T cells (Treg) suppress the proliferation of conventional T cells, maintaining peripheral tolerance. Surface markers used to define Treg are CD4, CD25 and CD127 and CD4^+^ CD25^+^ CD127^low^ cells express high levels of the master regulatory transcription factor, Foxp3 [1]. Most reports of suppression assays use peripheral blood-isolated Treg, or spleen-derived Treg in the case of murine studies, as these sources provide a high yield of lymphocytes [2], [3], [4]. Isolation of lymphocytes from other tissues is more complex, resulting in lower yields of viable cells [5] and access may be limited to small tissue biopsies. In our method, lymphocytes were isolated from human tissue by an enzymatic and mechanical method. Specific lymphocyte subsets were then isolated by cell sorting and a suppression assay was set up as detailed herein.

### Samples

Peripheral blood and tissue samples of CRC were obtained from patients undergoing a bowel resection as part of their treatment for CRC at the QEHB. Ethical approval had been obtained from the local research and ethics committee (LREC South Birmingham 2003/242, renewed 2012) and all patients donating tissue had given full prior consent.

### Lymphocyte isolation

1. CRC tissue samples were diced into small 2–3 mm pieces and placed in 10 ml of digestion buffer in a C tube (130-093-237, Miltenyi Biotec Ltd., UK). This buffer contained collagenase II (20 mg/ml, C2-22, VWR International Ltd., UK) and antibiotics (penicillin 1 U/ml, streptomycin 1 μg/ml, gentamicin 10 μg/ml, amphotericin B 0.25 μg/ml) in RPMI 1640 medium (31870-082, Life Technologies Ltd., UK).
2. The C tube was spun on the gentleMACS dissociator V1.02 (130-093-235, Miltenyi Biotec Ltd., UK) using the h_tumour_01.01 programme and incubated for 30 min at 37 °C with agitation. The C tube was spun on the gentleMACS dissociator h_tumour_02.01 programme and incubated at 37 °C for a further 30 min with agitation. Finally, the C tube was spun on the gentleMACS dissociator h_tumour_03.01 programme.
3. Digested tissue was passed through a cell strainer (352350, BD Biosciences Ltd., UK) and layered on top of a discontinuous gradient of 70% and 30% Percoll solutions (17-0891-01, GE Healthcare Ltd., UK) and centrifuged at 300 × *g* for 25 min.
4. The mononuclear cell band was aspirated and the cell suspension was washed and resuspended in 1 ml of RPMI 1640 media containing 1% foetal calf serum (FCS, 10270-106, Life Technologies Ltd., UK) and antibiotics, as above.
5. 10 ml peripheral blood from healthy volunteers was layered on top of an equal volume of Lymphoprep™ (07851, Stemcell Technologies Ltd., UK) and centrifuged at 300 × *g* for 25 min. The mononuclear cell band was aspirated, washed and resuspended in RPMI 1640 media containing 1% FCS.

### Lymphocyte labelling

1. The CRC tissue cell suspension was labelled with a fixable live/dead marker (L10119, Life Technologies Ltd., UK) at 5 μM concentration and incubated at 4 °C for 20 min.
2. Small volumes (50 μl) of the CRC tissue cell suspension were used for single fluorochrome labelling. The majority of the sample (700 μl) was labelled with the full antibody cocktail at 4 °C for 30 min, ready for cell sorting. The antibodies used to label the CRC tissue cell suspension were: anti-human CD4-PECy7 (317414, Clone OKT4, Biolegend), anti-human CD25-APC (555434, Clone M-A251, BD), CD127-FITC (560549, Clone HIL-7R-M21, BD) and anti-human CCR5-PE (FAB1802P, Clone CTC5, R&D Systems). The Treg population was defined as live CD4^+^ CD25^+^ CD127^low^ cells while conventional T cells (Tconv) were defined as live CD4^+^ CD25^−^ cells.
3. The remaining 100 μl of the sample was used for intra-cellular staining of Foxp3 to confirm the Treg phenotype and was first labelled with the cell surface antibodies: CD4-PECy7, CD25-APC and CD127-FITC (as above) and then fixed and permeabilised using the Foxp3/Transcription Factor Staining Buffer Set (0-5523-00, eBioscience), according to the manufacturer's instructions. The permeabilised cell suspension was divided into two equal volumes: half was labelled with anti-human Foxp3-PE (12-4776-42, eBioscience) and the other half with isotype-matched control antibody (12-4321-42, eBioscience) at 4 °C for 30 min. These samples were analysed by flow cytometry following colour compensation using single fluorochrome-labelled samples, allowing assessment of Foxp3 expression of CD4^+^ CD25^+^ CD127^low^ cells (see Graphical Abstract).
4. Peripheral blood isolated mononuclear cells were labelled with CellTrace Violet dye (C34557, Life Technologies Ltd., UK) at 37 °C for 20 min and washed twice. The cell suspension was labelled with CD4-PECy7 and CD25-APC antibodies, as above. CellTrace Violet-labelled CD4^+^CD25^−^ cells are referred to as responder T cells (Tresp).

### Cell sorting

1. Cell sorting was used to add Treg or Tconv to a 96-well plate followed by Tresp. The Treg population was defined by gating on live CD4^+^ CD25^+^ CD127^low^ cells while Tconv were defined by gating on live CD4^+^ CD25^−^ cells (see Graphical Abstract).
2. Each well of a round-bottomed 96-well plate was filled with 100 μl of RPMI + 10% FCS. An equal number of CCR5^low^ Treg, CCR5^high^ Treg and Tconv were 3-way sorted from labelled CRC-isolated cell suspension into separate wells using a MoFlo XDP High-Speed Cell Sorter (Beckman Coulter Inc., USA) in purity mode. In some cases, sorted lymphocytes were analysed on a flow cytometer to assess purity (see Fig. 1). Typical yields were between 10 000 and 60 000 Treg, according to the sorter event counter. In cases with high yields, 10 000 CCR5^low^ Treg, CCR5^high^ Treg and Tconv were 3-way sorted into separate Eppendorfs containing 1 ml of PBS in preparation for later DNA extraction and analysis of the TSDR (Treg-specific demethylated region).

**Fig. 1:**
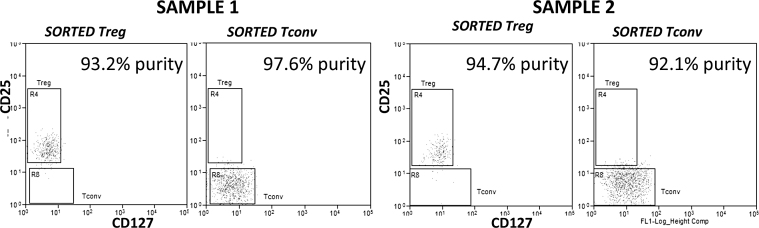
Purity check of sorted Treg and Tconv from two samples. Percentages in top-right of histogram represent the purity of sorted Treg subsets.
3. Violet-labelled Tresp (peripheral blood-isolated CD4^+^CD25^−^ cells) were plate-sorted into the same wells as the previously sorted Treg and Tconv, yielding Treg/Tconv:Tresp ratios of 1:1, 1:2 or 1:4.

### Suppression assay

Human Treg Suppression Inspector beads (Miltenyi Biotec Ltd., UK) provided a polyclonal stimulus for proliferation and were added to each well at a bead:lymphocyte ratio of 1:2. The total volume of each well was made up to 200 μl with RPMI 1640 media + 10% FCS. The plate was incubated at 37 °C and 5% CO_2_ for 3 days. Violet dye wash-out was analysed by flow cytometry (Cyan ADP flow cytometer, Beckman Coulter). Violet-labelled cells cultured alone, in the absence of bead stimulation, were used as a negative control, enabling a gate to be set around the proliferating cell population. The percentage of Tresp proliferating is thus easily determined. However a small number of proliferating Tresp undergoing multiple divisions will have the effect of under-representing the suppressive capacity of Treg, an effect that can be overcome by measuring the division index (DI). The DI is defined as the average number of divisions for all Tresp in the culture and was calculated using Flowjo version 8.7 (Tree Star Inc.) [6]. Results were reported as percent suppression [3]:

$$\begin{array}{l} \text{Percent}\;\text{suppression}=100-\frac{\text{Percentage}\;\text{of}\;\text{proliferating}\;\text{cells}\;(\text{or}\;\text{DI})\;\text{with}\;\text{Treg}\;\text{present}}{\text{Percentage}\;\text{of}\;\text{proliferating}\;\text{cells}\;(\text{or}\;\text{DI})\;\text{without}\;\text{Treg}\;\text{present}}\times 100 \end{array}$$

Differences in percent suppression, calculated using both percentage of proliferating cells and DI, between CRC-isolated Treg and CD4^+^CD25^−^ cells were tested for statistical significance using the Wilcoxin signed-rank test.

Representative results from one experiment, in which enough Treg were isolated to perform three different Treg:Tresp ratios, are shown in Fig. 2. Following 72 h of culture, 75% of Tresp proliferated in response to Treg Suppression Inspector beads alone. Both CRC-isolated CCR5^low^ Treg and CCR5^high^ Treg suppressed proliferation of Tresp in a dose-dependent manner, while CRC-isolated Tconv did not. The differences in percent suppression by CCR5^low^ Treg, CCR5^high^ Treg and Tconv were compared using pooled data for Treg:Tresp ratios of 1:1 and 1:2 over a series of five experiments (see Table 1). Both CCR5^low^ Treg and CCR5^high^ Treg suppressed Tresp proliferation significantly more then Tconv, *P* = 0.043 and *P* = 0.018, respectively. Also, CCR5^high^ Treg suppressed Tresp proliferation significantly more than CCR5^low^ Treg (*P* = 0.018), an effect that has been noted previously in patients with psoriasis [7].

**Fig. 2:**
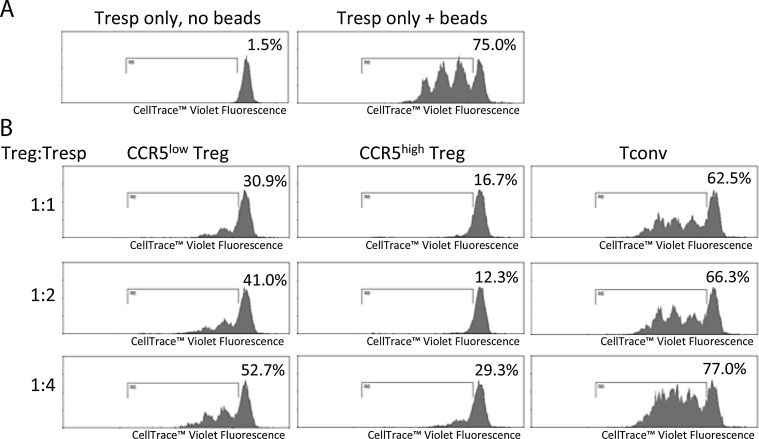
Suppression assay using CCR5^low^ and CCR5^high^ Treg isolated from one sample of CRC. (A) Tresp proliferation following 72 h of culture with or without Treg Suppression Inspector beads. (B) Tresp proliferation following 72 h of culture with Treg Suppression Inspector beads and either CRC-isolated CCR5^low^ Treg, CCR5^high^ Treg or Tconv. Numbers at the top-right of each histogram indicates the percentage of proliferating cells.

**Table 1:** Suppression assay summary.

Experiment	Ratio Treg:Tresp	Number Treg/Tconv	Percent suppression using the percentage of proliferating cells (%)			Percent suppression using the Divison Index (%)		
			CCR5^low^ Treg	CCR5^high^ Treg	Tconv	CCR5^low^ Treg	CCR5^high^ Treg	Tconv
A	1:1	10 000	28.5	32.1	0.0	82.0	87.4	37.1
B	1:1	5000	75.1	81.4	−16.0	96.1	95.9	44.9
C	1:1	10 000	58.8	83.6	16.7	75.2	88.1	46.6
D	1:1	10 000	6.7	46.6	−1.3	28.8	61.3	−2.7
C	1:2	5000	45.3	77.7	11.6	85.1	91.8	39.1
D	1:2	5000	8.7	34.3	16.8	37.8	74.8	−34.2
E	1:2	5000	55.1	72.2	−0.7	85.8	92.8	4.1
C	1:4	2500	29.7	60.9	−2.7	61.5	85.1	16.8
D	1:4	2500	0.0	6.0	−9.4	3.6	28.8	0.9
Pooled data for ratios 1:1 and 1:2			45.3	72.2	−0.7	82.0	88.1	37.1
Median (interquartile range)			(18.6–56.9)	(40.4–79.6)	(−2.1 to 14.1)	(56.5–85.5)	(81.1–92.3)	(69.2–42.0)

Percent suppression method	CCR5^low^ Treg versus Tconv	CCR5^high^ Treg versus Tconv	CCR5^low^ Treg versus CCR5^high^ Treg
Percentage of proliferating cells	0.043	0.018	0.018
Division index	0.018	0.018	0.028

The percent suppression of Tresp proliferation is shown for CRC-isolated CCR5^low^ Treg, CCR5^high^ Treg and Tconv at different Treg:Tresp ratios. The number of Treg/Tconv represents the number of sort events given by the MoFlo XDP High-Speed Cell Sorter, rather than the absolute number of sorted viable cells.

The differences in percent suppression for ratios of 1:1 and 1:2 between different cell populations were tested for statistical significance using the Wilcoxon signed-rank test (*P*-values shown below).

### TSDR analysis

TSDR analysis was performed on sorted Treg to show that Treg isolated from human tissues were naturally-occurring Treg as opposed to induced Treg.

1. Eppendorfs containing sorted Treg and Tconv were pelleted by centrifugation in a microfuge at 300 × *g* for 10 min then resuspended in 200 μl of PBS. DNA was extracted from the cell suspensions using the DNeasy Blood & Tissue Kit (69504, Qiagen) according to the manufacturer's instructions. DNA samples were bisulphite treated using the Epitect Bisulfite kit (59104, Qiagen) and the quantity and quality of recovered bisulphite-treated DNA was assessed by measurement of UV absorbance at 260/280 nm.
2. The TSDR region was amplified using primers designed to amplify the TSDR region irrespective of its methylation status. A Taqman^®^ probe labelled with 6-carboxyfluorescein (FAM) was designed to specifically bind to an unmethylated sequence while another Taqman^®^ probe labelled with 4,7,2′-trichloro-7′-phenyl-6-carboxyfluorescein (VIC) was designed to specifically bind to a methylated sequence. To achieve maximum specificity, each probe was designed to span 3 CpGs within the TSDR. The primers, probes and method have been previously published [8]. Primers and probes were obtained from Applied Biosystems Inc., USA.
3. The following programme was run using the LightCycler 480:

**Table:** 

Pre-incubation	1 cycle – 95 °C for 10 min
Amplification	55 cycles – 95 °C for 15 s; 60 °C for 60 s
Colour compensation	1 cycle – 95 °C for 10 s; 40 °C for 30 s
	85 °C, continuous acquisition mode, 5 acquisitions per 1 °C
Cooling	1 cycle – 40 °C for 10 s

4. VIC and FAM were colour-compensated using the LightCycler 480 software followed by absolute quantification of unmethylated and methylated target expression. The average methylated *Ct* was subtracted from the average unmethylated *Ct* to yield a ∂*Ct* value. The percentage of unmethylated DNA was estimated by plotting the ∂*Ct* value on a published standard curve [8].
5. Analysis of the demethylation status at the TSDR for CCR5^low^ Treg, CCR5^high^ Treg and Tconv, isolated from four different CRC samples, revealed a significantly greater percentage of unmethylated DNA at the TSDR for both CCR5^low^- and CCR5^high^ Treg compared with Tconv (see Fig. 3).

**Fig. 3:**
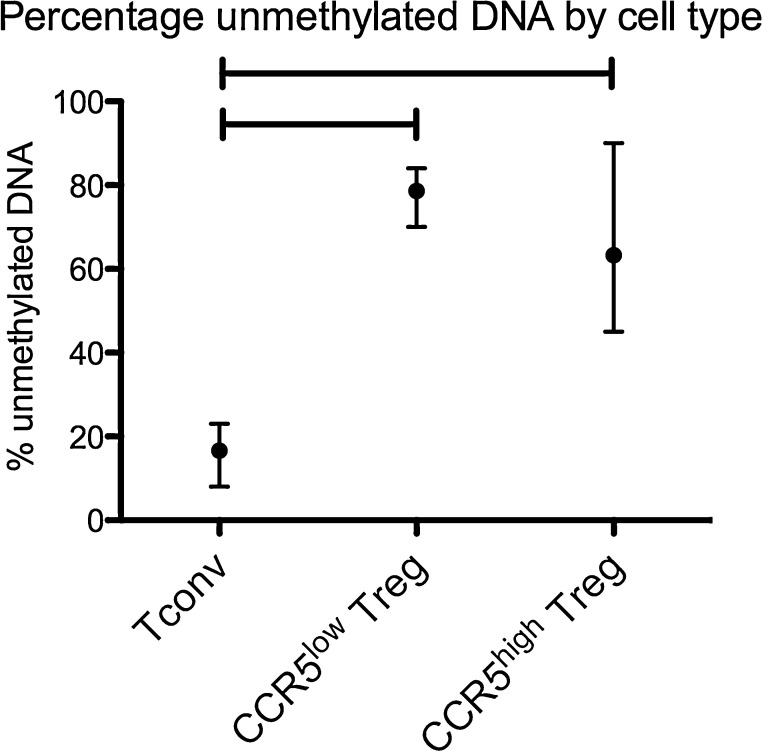
Percentage of unmethylated DNA at the TSDR by cell type. The mean percentage of unmethylated DNA is plotted for Tconv, CCR5low Treg and CCR5high Treg, isolated from four different colorectal cancer samples. Error bars represent the range of percentage of unmethylated DNA. Capped lines represent a statistically significant difference (Wilcoxon signed-rank test). Sorted Treg are naturally-occurring thymic emigrants.

## Author contributions

STW was the primary researcher who developed, executed and analysed the performed experiments. STW drafted the manuscript. KKL assisted in obtaining and analysing data. KKL and SMC edited the manuscript.

## Figures and Tables

**Figure:**