# Supplementary material for: A method for conducting suppression assays using small numbers of tissue-isolated regulatory T cells
Source: MethodsX. 2014 Aug 28;1:168–74. doi: 10.1016/j.mex.2014.08.012 (PMC4340526; doi:10.1016/j.mex.2014.08.012)
Supplement: Supplementary file 1 [file mmc1.docx]

Regulatory T cells (Treg) suppress the proliferation of conventional T cells, maintaining peripheral tolerance. Surface markers used to define Treg are CD4^,^ CD25 and CD127 and CD4^+^CD25^+^CD127^low^ cells express high levels of the master regulatory transcription factor, Foxp3 (1). With regard human Treg, suppressive capacity is limited to the CD25^bright^ population (2). The first in vitro suppression assay was reported in 1998 (3) and has become a commonly performed assay in immunology-based laboratories around the world. The assay is relatively simple to perform, is reproducible and can be used in conjunction with genetically-altered cells or inhibitors to determine important components of the suppression mechanism (4).

Most reports of suppression assays use peripheral blood-isolated Treg, or spleen-derived Treg in the case of murine studies, as these sources provide a high yield of lymphocytes (4–6). Isolation of lymphocytes from other tissues is more complex, resulting in lower yields of viable cells (7) and access may be limited to small tissue biopsies. In addition, the increasing recognition of highly suppressive Treg subsets demands optimization of current suppression assay protocols. It is possible to perform suppression assays with small numbers of Treg, although published protocols involve knowing the absolute number of isolated cells - a technique which requires a minimum of 100 000 cells for accurate counts using a hemocytometer. It is therefore desirable to sort lymphocytes directly into a 96-well plate for use in suppression assays by fluorescent-activated cell sorting (FACS), thereby maximizing the lymphocyte yield. The sorter event count can be used as a guide to the number of lymphocytes sorted into the well. There are however some technical considerations to this method that need to be addressed in order to perform a successful reproducible suppression assay. A method that was used successfully, involving the isolation of Treg subsets from human colorectal cancer (CRC) tissue and assessing their ability to suppress the proliferation of allogeneic peripheral blood responder T cells (CD4^+^CD25^-^, Tresp), is presented in this article.

This method has proven useful in situations where functional assays are required using specific subpopulations of cells. To emphasise the usefulness of the method, suppressive capacity of CCR5^high^ Treg was compared with that of CCR5^low^ Treg. Interestingly, CCR5^high^ Treg demonstrated enhanced suppression, an effect that has been noted previously in patients with psoriasis (8).

The method involved cell sorting using a tight gating strategy for the Treg markers CD127 and CD25 and ensuring sorting of live cells by use of a live/dead marker (see Graphical Abstract). The CD127 – CD25 dot plot for peripheral blood isolated lymphocytes is shown for comparison in Supplementary Figure 1. Tresp were then plate-sorted into appropriate wells having been pre-labelled with CellTrace Violet. Proliferation of Tresp was always detected but initially, suppression of proliferation by Treg was not observed. According to the Treg Suppression Inspector manufacturer’s protocol, the beads should be added at a bead:lymphocyte ratio of 1:1. However, after repeated suppression assays in which there was cell proliferation but no suppression, the ratio was changed to 1:2. This allowed both proliferation and suppression to be detected. The likely explanation for this relates to the absolute number of viable cells sorted into the plate. For example, the sorter is instructed to sort 10,000 Treg into a well followed by 10,000 Tresp. Following this sort, 20,000 beads would be added to the co-culture at a bead:lymphocyte ratio of 1:1. However, the sorter is only able to report sort *events* - the true number of viable cells recovered is significantly less due to cell death occurring pre- or post-sort; adherence of cells to tube walls; precision of the flow cytometer. Typically, 50% recovery is obtained (9). Therefore, the actual bead:lymphocyte ratio is in the order of 2:1 and this polyclonal stimulus for proliferation may be too strong for Treg to suppress. The typical true lymphocyte yield post-sort can be measured by sorting a set number of cells and then evaluating the recovery by flow cytometry. The importance of the cell:bead ratio has been previously reported, demonstrating that the bead concentration can affect the ability of Treg to suppress T cell proliferation in vitro (10).

Due the low number of Treg isolated from the tumor, the suppression assay protocol was optimized for low numbers of Tresp compared with traditional protocols. In our hands, we were able to reduce the number of Tresp to 10,000 cells compared with the usual 50,000 cells without compromising the accuracy of the assay (see figure 1b). In addition, due to the importance of cell-contact dependent suppression, round-bottomed well-plates were used to maximize the suppression by Treg in a low yield scenario.

Various methods have been used to provide a polyclonal stimulus for T cell proliferation in suppression assays. Irradiated splenocytes or purified dendritic cells can be combined with soluble anti-CD3 to provide a more physiological stimulus for proliferation (4). Addition of a third population of cells into the assay does however introduce more variability. Plate-bound or soluble anti-CD3 and anti-CD28 may be used as a stimulus, although this method does not permit calibration of the strength of the proliferation signal (11). Beads loaded with anti-CD3 and anti-CD28 antibodies can be titrated to the desired proliferative response (4) and in our described method Treg Suppression Inspector beads (Miltenyi Biotec Ltd, UK) were favored. The cell:bead ratio may need to be adjusted according to the post-sorting true cell yield.

The classical suppression assay measured ^3^H-thymidine incorporation into proliferating cells (3). However, this technique may underestimate Treg-dependent suppression as ^3^H-thymidine is also incorporated into proliferating Treg (12). Fluorescent-labeling of Tresp permits the independent proliferation of Tresp to be measured and multiple divisions to be visualized. This was evident even with the small number of Tresp used in this study (see figure 3).

In summary, the described method can be used to demonstrate suppression of allogeneic T cell proliferation by small numbers of tissue-isolated Treg. A live/dead marker and appropriate strict gating should be used to ensure Treg purity. It is suggested that Foxp3 expression should be measured in isolated Treg to support this. Sort purity checks should be performed as well as a true cell yield measurement. Finally, beads are recommended to provide a polyclonal stimulus for proliferation and should be titrated to response.

**References**

1	Liu W, Putnam AL, Xu-yu Z, Szot GL, Lee MR, Zhu S, *et al.* CD127 expression inversely correlates with FoxP3 and suppressive function of human CD4+ T reg cells. *J Exp Med* 2006; **203**: 1701–11.

2	Baecher-Allan C, Wolf E, Hafler DA. Functional analysis of highly defined, FACS-isolated populations of human regulatory CD4+CD25+ T cells. *Clin Immunol* 2005; **115**: 10–8.

3	Thornton AM, Shevach EM. CD4+CD25+ immunoregulatory T cells suppress polyclonal T cell activation in vitro by inhibiting interleukin 2 production. *J Exp Med* 1998; **188**: 287–96.

4	Collison LW, Vignali DAA. In Vitro Treg Suppression Assays. *Methods Mol Biol Clifton NJ* 2011; **707**: 21–37.

5	McMurchy AN, Levings MK. Suppression assays with human T regulatory cells: A technical guide. *Eur J Immunol* 2012; **42**: 27–34.

6	Quah BJC, Parish CR. New and improved methods for measuring lymphocyte proliferation in vitro and in vivo using CFSE-like fluorescent dyes. *J Immunol Methods* 2012; **379**: 1–14.

7	Zhang J, Dong Z, Zhou R, Luo D, Wei H, Tian Z. Isolation of lymphocytes and their innate immune characterizations from liver, intestine, lung and uterus. *Cell Mol Immunol* 2005; **2**: 271–80.

8	Soler DC, Sugiyama H, Young AB, Massari JV, McCormick TS, Cooper KD. Psoriasis patients exhibit impairment of the high potency CCR5(+) T regulatory cell subset. *Clin Immunol* 2013; **149**: 111–8.

9	Arnold LW, Lannigan J. Practical Issues in High-Speed Cell Sorting. In *Current Protocols in Cytometry*: John Wiley & Sons, Inc., 2001 (http://onlinelibrary.wiley.com/doi/10.1002/0471142956.cy0124s51/abstract).

10	Oberg H-H, Wesch D, Lenke J, Kabelitz D. An optimized method for the functional analysis of human regulatory T cells. *Scand J Immunol* 2006; **64**: 353–60.

11	Schneider A, Buckner JH. Assessment of Suppressive Capacity by Human Regulatory T Cells Using a Reproducible, Bi-Directional CFSE-Based In Vitro Assay. In *Regulatory T Cells* (eds G Kassiotis, A Liston): 233–41. Humana Press, 2011.

12	Venken K, Thewissen M, Hellings N, Somers V, Hensen K, Rummens J-L, *et al.* A CFSE based assay for measuring CD4+CD25+ regulatory T cell mediated suppression of auto-antigen specific and polyclonal T cell responses. *J Immunol Methods* 2007; **322**: 1–11.
